# Giant Cell Tumor of Extensor Tendon Sheath in Ring Finger: A Case Report

**DOI:** 10.7759/cureus.29605

**Published:** 2022-09-26

**Authors:** Shivshankar Jadhav, Abhiram Awasthi, Sanjay Deshpande, Vivek Jadawala, Ankur Salwan

**Affiliations:** 1 Department of Orthopedics and Traumatology, Datta Meghe Institute of Medical Sciences, Wardha, IND

**Keywords:** synovioma, myeloid epithelioma, excisional biopsy, tendon sheath, giant-cell tumor

## Abstract

Giant cell tumour of the tendon sheath (GCTTS) is a rare benign soft tissue tumour with no known cause. It is more prevalent in the hand than in the ankle and foot. It appears as a non-painful, perceptible enlargement. Although pre-operative imaging and fine-needle aspiration cytology (FNAC) corroborate suspicion, histology following surgical resection is used to confirm the diagnosis. Due to its rarity, a case of giant cell tumour (GCT) of the extensor tendon sheath of the left ring finger is reported here. A 39-year-old female presented with a six-month history of painless swelling over left ring finger. The swelling was spontaneous, slowly progressive and painless. On clinical examination, a 1.5 cm x 1 cm firm swelling was seen on the dorsal surface of the left ring finger extending from the distal portion of the middle phalanx to the proximal half of the distal phalanx. The swelling was well-defined, smooth, firm, and uniform in consistency. The swelling was movable sideways with no attachment to the bone when examined clinically. X-ray of the hand showed soft tissue mass without the involvement of the bone. Soft tissue mass was seen on ultrasonography. An excisional biopsy was done. Histopathology showed typical features of GCTTS. Our case is a rare example of GCTTS in a single digit of the hand. Furthermore, considering its high recurrence risk, the tumour should be totally excised. Finally, if required, the hand's function should be recreated to minimise the loss.

## Introduction

Giant cell tumour of the tendon sheath (GCTTS) is a benign soft tissue tumour that appears as a solitary, extra-articular, and firm swelling that lasts for years. Overall the prevalence is one in 50,000 people, and it primarily affects women between the ages of 30 and 50, adults. It is more prevalent in females with a female-to-male ratio of 3:2 [1]. It primarily affects tiny joints in the hands and feet, but it can also be observed elsewhere like ankle, knee, elbow, and hip. Excision of the entire local area with or without radiation therapy is the preferred modality of treatment. In 10-20% of cases, local recurrence has been recorded.

Fibrous xanthoma was the name given to the condition by Chassaignac in 1852 [2]. Fibro-histiocytoma of synovium, pigmented nodular synovitis, tenosynovial giant cell tumour (GCT), localised nodular tenosynovitis, benign synovioma, sclerosing hemangioma, myeloid epithelioma, fibrous xanthoma, giant-cell fibro-hemangioma, and fibrous xanthoma are other names given to the tumour [2,3]. GCTTS is still debated as a tumour or a bulk of reactive proliferation [4]. About 15% of patients describe some form of trauma prior to swelling; however, there is no evidence that this is the cause [5].

It is divided into two types based on clinical and biological manifestations: localized and diffuse forms. These can also be intra-articular and extra-articular. The benign localized type affects the hand and fingers, but the more aggressive diffuse form affects major joints [6]. It was formerly thought to be an inflammatory swelling; however, aneuploidy and clonal chromosomal aberrations have been discovered in certain instances, suggesting a neoplastic origin [7].

GCTTS is most commonly found around the index or long finger's distal interphalangeal joint followed by ring and little finger. The following case presents with the features suggesting GCTTS of ring finger [8].

## Case presentation

A 39-year-old female patient came to the hospital with a six-month history of painless swelling over left ring finger. The swelling was spontaneous with no history of trauma. It gradually increased in size, hampering the activities of daily living of the patient.

**Figure 1:**
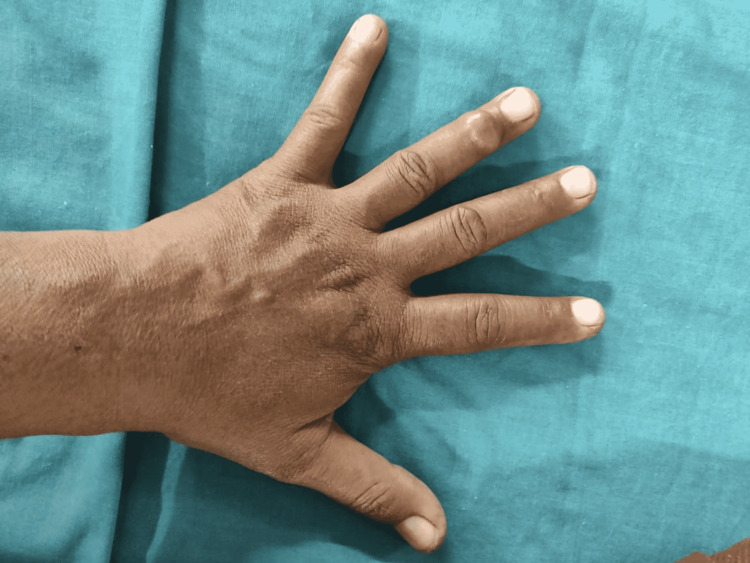
Swelling on dorsal aspect of ring finger

On clinical examination, a 1.5 cm x 1 cm firm swelling was seen on the dorsal surface of the left ring finger (Figure 1), extending from the distal portion of the middle phalanx to the proximal half of the distal phalanx. There was no localized temperature or skin involvement. The swelling was well defined, smooth, firm and uniform in consistency. The swelling was movable sideways with no attachment to the bone when examined clinically. It adhered to the underlying soft tissue and hence moved with the movement of the finger. Normal capillary circulation was present. Paresthesia was not noted.

**Figure 2:**
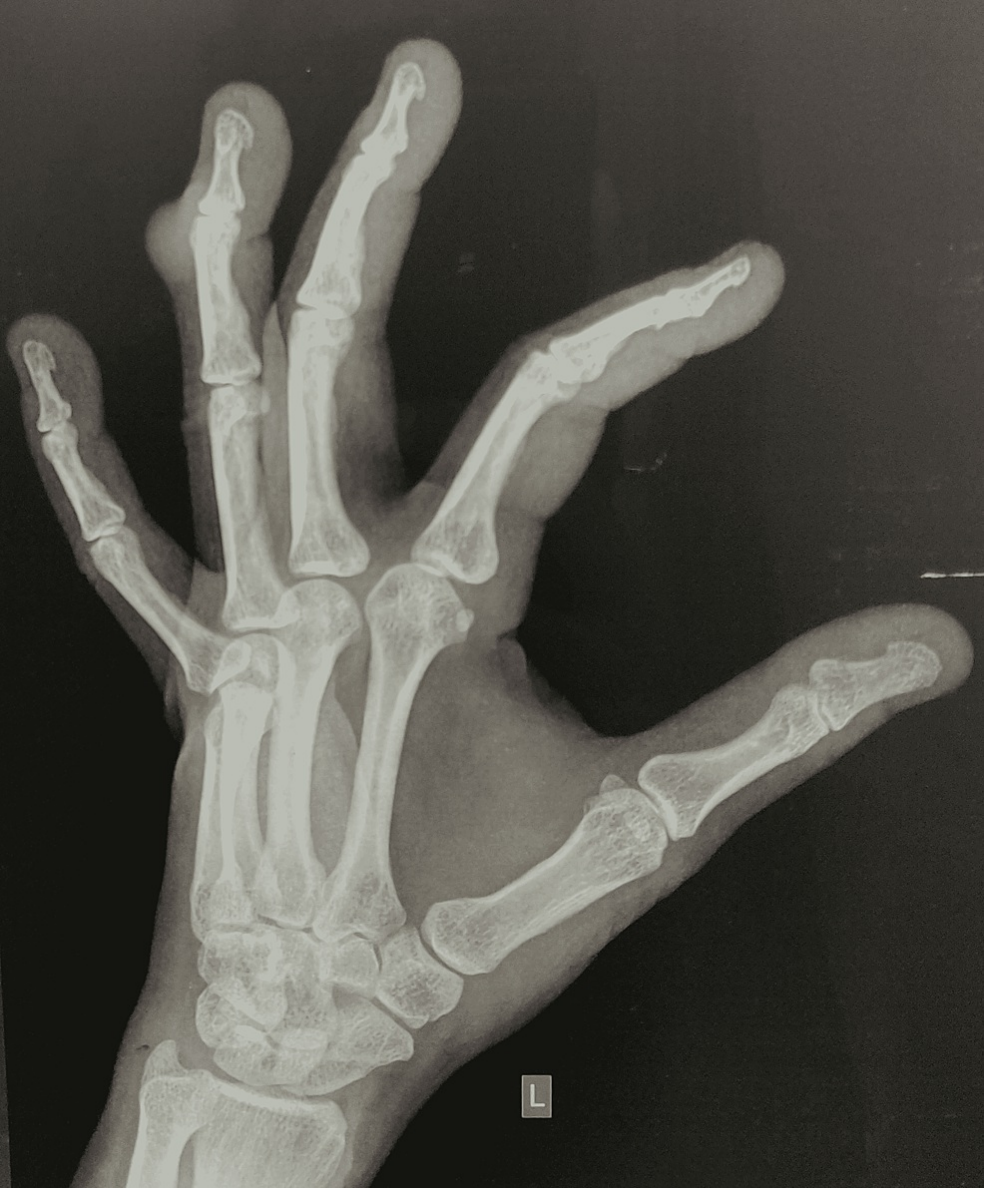
X-ray of left hand showing soft tissue shadow in ring finger

X-ray anteroposterior (AP) and oblique view of left hand (Figure 2) showed soft tissue shadow over distal portion of the middle phalanx to the proximal half of the distal phalanx with no bone involvement. Ultrasonography was done which revealed mass attached to the underlying soft tissue on the dorsal surface of distal phalanx of left ring finger. Other investigations like complete blood counts, random blood sugar, liver function tests, and renal function tests were normal.

**Figure 3:**
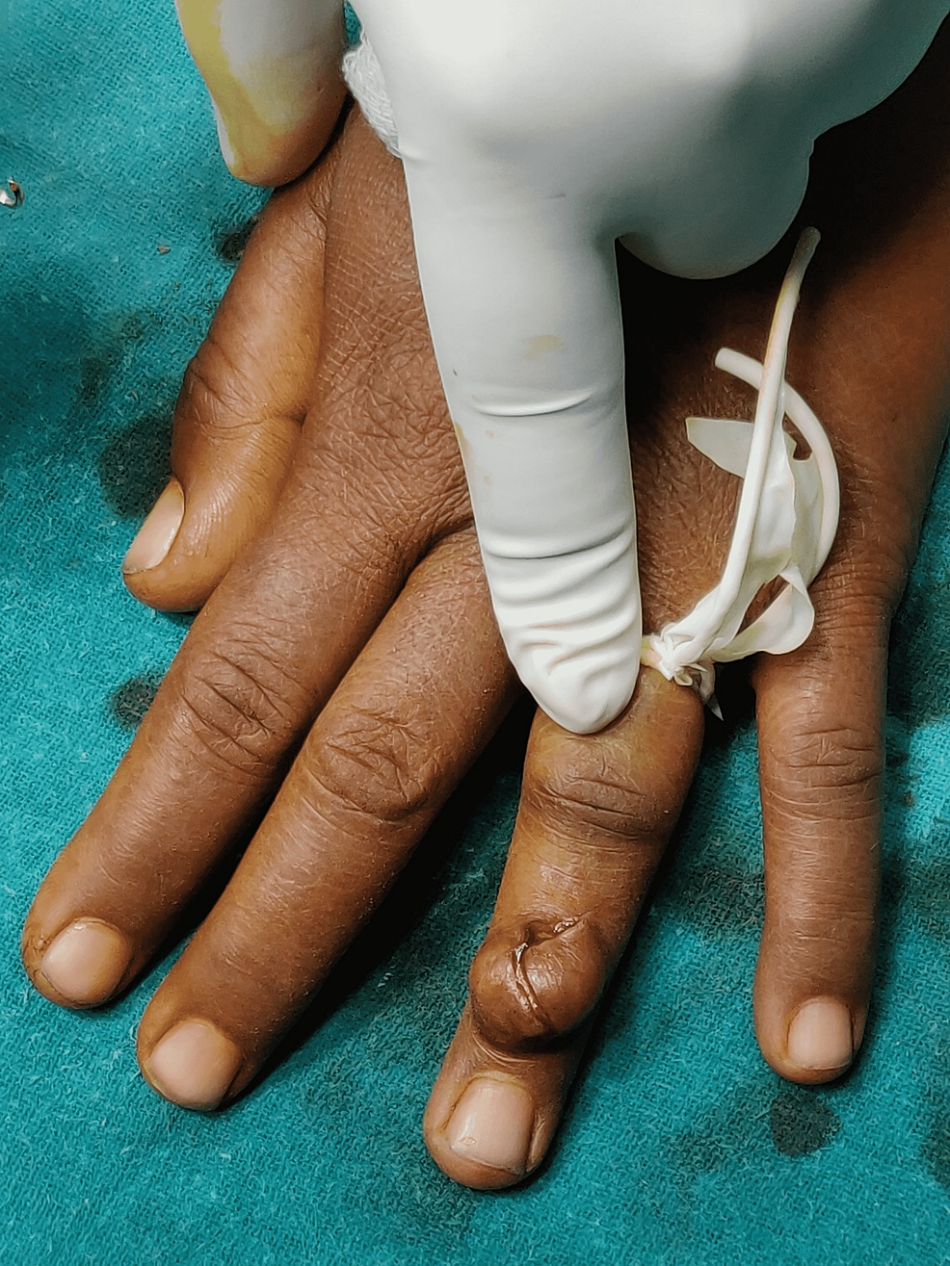
Curvilinear incision over the swelling

**Figure 4:**
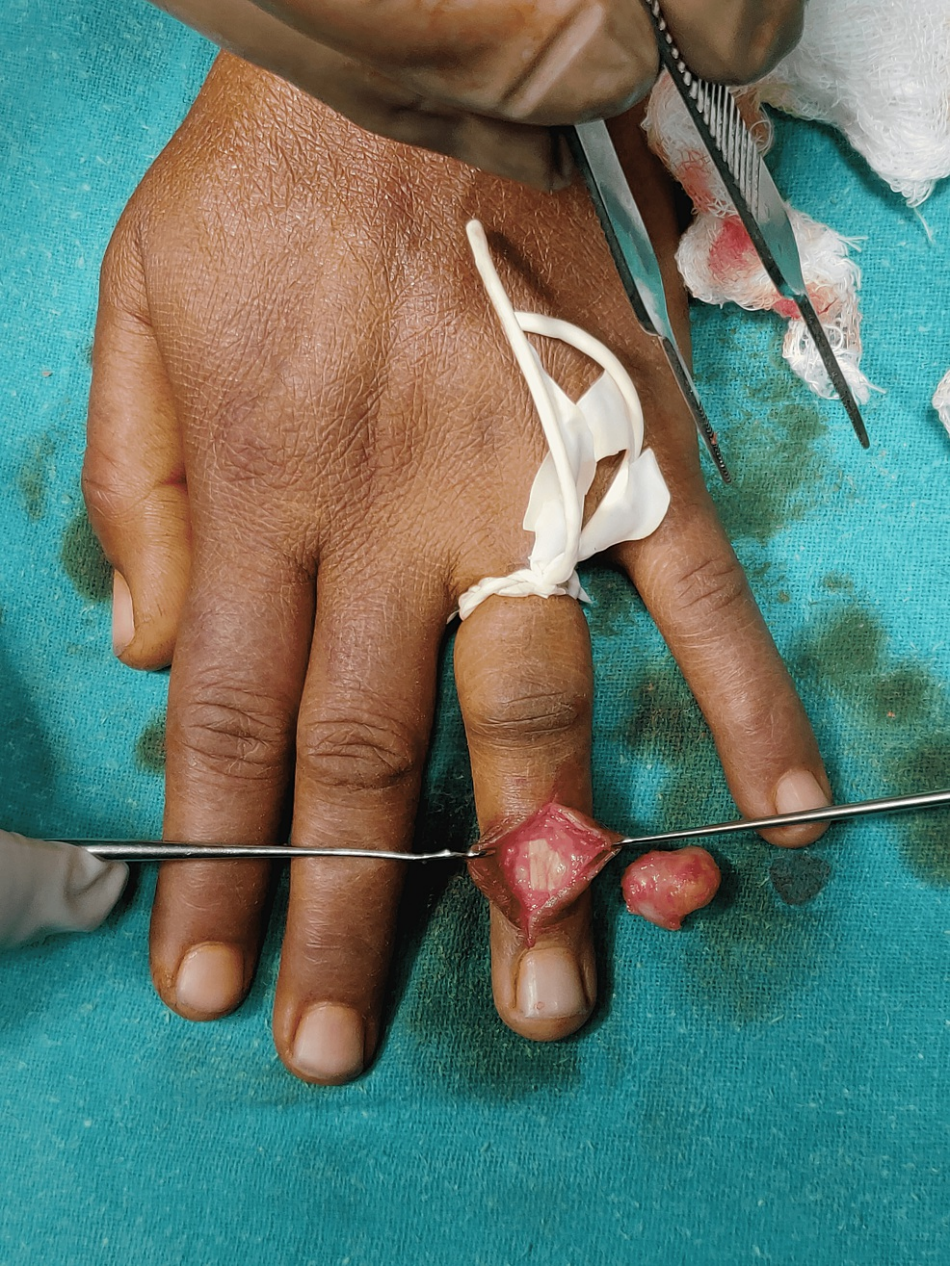
Tumour excised completely

The patient was surgically fit and an excisional biopsy of the underlying swelling was performed. It was done under local anaesthesia and sedation. Cutting the little finger of the sterile gloves, tourniquet was prepared and used for the surgery. A curvilinear incision was taken over the swelling (Figure 3). Soft tissue dissection was done and the tumour was seen adhered to the underlying extensor digitorum tendon of ring finger. Excision of tumour was easy and was excised completely (Figure 4). The closure was done in layers and the tourniquet was released.

**Figure 5:**
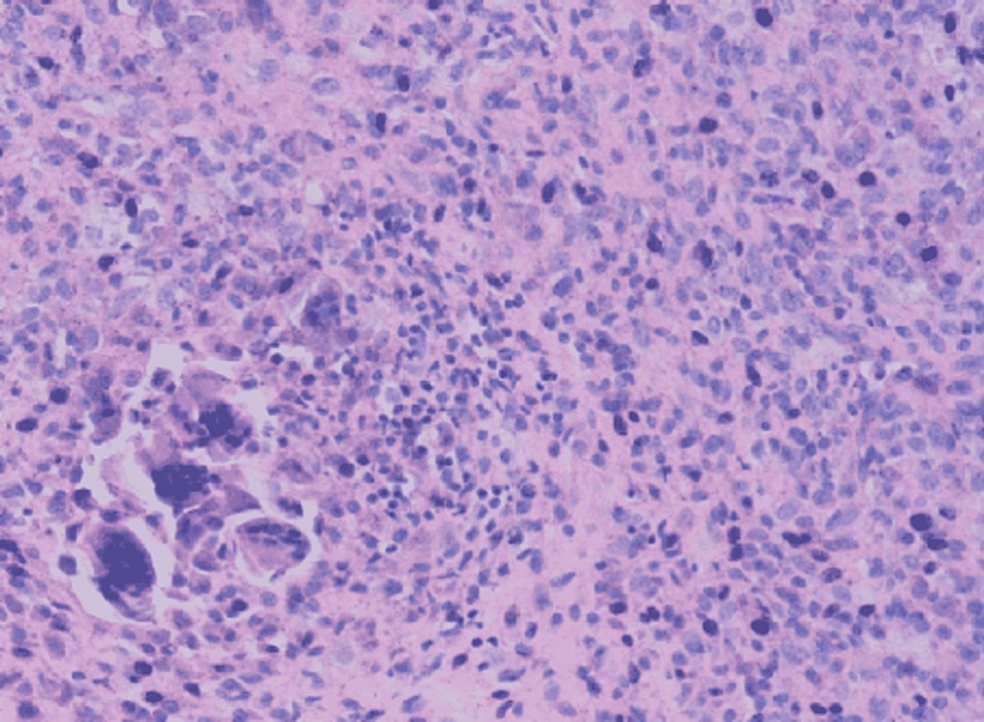
Histopathological examination showing multi-nucleated giant cells with histiocytes surrounded by fibro-fatty tissue (hematoxylin and eosin stain - magnification 200x)

The excised tumour was sent for histopathological examination, which showed polygonal to round histiocytes surrounded by multinucleated giant cells, fibro-fatty tissue (Figure 5) suggestive of GCTTS. Postoperatively follow-up was held every six months, and a full recovery was noticed without any significant restriction of movement of the finger. The patient was able to perform her activities of daily living. No evidence of recurrence was noted on clinical and radiological examination.

## Discussion

GCTTS is a benign fibrous tissue tumour that starts in the teno-synosheath, bursae, and synovium of the joint [9]. This tumour is more frequent in individuals between the ages of 30 and 50, and it is more common in women. The cause of GCTTS is unknown; however, it might be linked to an inflammatory response, a local lipid metabolic issue, or osteoclastic growth, infection or trauma [10].

GCTTS is mostly not adherent to the overlying structures but if it is big in size, maybe due to its prolonged duration, it may cause dent on the underlying bone. Patients present in the hospital late as this does not result in any functional disability. It is most commonly seen in hands but may also be seen in the foot and ankle [1-3]. In hands, it is seen commonly on the flexor aspect but in foot and ankle, it is seen on the lateral aspect of the dorsum, commonly the extensor tendons. As reported by Kant et al. after reviewing 26 cases, the affected age group was 21 to 40 years [1]. As per the study GCTTS affecting the bone shows two clinical presentations: nodular or localized type and diffuse type. Out of these two, localized or nodular type is mostly seen in hands while the diffuse type in joints.

As reported by Levi and Crafton, MRI has a crucial role in diagnosing GCTTS early [11]. On T1 and T2 weighted images, they are visualized as low signal intensity, with its actual dimensions, its extent, and further assisting in the surgical approach. However, MRI cannot distinguish among GCTTS, synovial chondromatosis, and villonodular synovitis. Therefore, histopathological examination is necessary.

Recurrence and damage to the joints are the highest risk of GCTTS that require resection of tumour surgically [12]. A recurrence rate of seven to forty-four percent was reported by Williams et al., 15-45% by Choughri et el., and six percent by Hakan et al. [13-15]. Factors that predict the recurrence include tumour located at interphalangeal joints, stress erosions on radiographs, osteoarthritic changes, and incomplete excision of tumour. Lower recurrence rates have been noted if the surgeons use operating microscopes. In our study, the lesions on tendon sheaths have been excised completely to prevent any recurrence. Thus, radical excision as surgical management plays an important role in preventing recurrence. As suggested by Kotwal et al. after reviewing 14 cases, the use of radiation therapy of 20 Gy in doses of 2 Gy daily has shown zero percent recurrence [16]. However, the roles of radiation therapy and tumour histology stay controversial and inconclusive.

The case reported in this article presented to the hospital as a benign tumour which was diagnosed and confirmed on histo-pathological examination after excision of tumour surgically. At six months follow-up, the patient showed no ultrasound, clinical, or radiological evidence of recurrence. In addition, a minimum of two years of follow-up is planned to detect any recurrence.

## Conclusions

Our case is an example of GCTTS in a single digit of the hand. Furthermore, considering its high recurrence risk, the tumour should be totally excised. Although FNAC followed by excisional biopsy is diagnostic and curative, the patient should be monitored for recurrence detection and treatment. Additionally, a proper radiographic and histopathological examination is to be done on the tumour to rule out whether it's malignant or benign, which may or may not require any further treatment. Finally, if required, the hand's function should be recreated to minimize the loss.
